# From Several Puck-like Inter-Fiber Failure Criteria to Longitudinal Compressive Failure: An Extension and Application for UD Composites

**DOI:** 10.3390/polym17121613

**Published:** 2025-06-10

**Authors:** Jiongyao Shen, Zhongxu Liu, Junhua Guo

**Affiliations:** School of Energy and Power, Jiangsu University of Science and Technology, Zhenjiang 212100, China

**Keywords:** UD composites, longitudinal compressive failure, failure criteria, inter-fiber failure, fiber kinking failure

## Abstract

The LaRC02 criterion is a classical criterion for determining fiber kinking failure of UD laminates under longitudinal compression (LC), but its basis for determining matrix cracking in a fiber kinking coordinate system is based on stress-invariant theory rather than on a physical mechanism. Herein, three Puck-like physical-mechanism-based inter-fiber failure criteria are extended to LC failure of UD composites, and thus three failure criteria (denoted as LC-Guo, LC-Li, and LC-Puck failure criteria) are constructed for fiber kinking failure determination. The stresses in the global coordinate system are transformed to the fiber kinking coordinate system by a three-level coordinate system transformation, and then the failure determination is performed using the three Puck-like criteria. The results show that the overall accuracy of the three proposed criteria is higher than that of the LaRC02 criterion, especially the LC-Guo criterion. Additionally, an analysis of the influence of material properties shows that the failure envelope curves tend to be conservative, and the predicted off-axial compression strength decreases as the transverse compression strength and in-plane shear strength increase and the transverse tensile strength decreases. This work proposes a more reasonable assessment methodology for the determination of LC failure of UD composites, which has important theoretical significance and engineering value.

## 1. Introduction

Fiber-reinforced composites are extensively applied in aerospace, automotive, marine, wind power, and other fields for their high specific stiffness/strength, superior fatigue resistance, and strong corrosion resistance [1,2,3,4]. To fully utilize the advantages of composite structures, it is necessary to establish rational failure criteria for strength design. Numerous scholars studied the failure theories of composites, and multifarious theories have sequentially demonstrated their respective advantages and limitations [5,6,7,8]. Among these theories, the fiber longitudinal compression (LC) failure modes are relatively understudied compared to the inter-fiber failure (IFF) modes, and their failure initiation and evolution are often simplistically defined and described [9]. The academic community has not yet fully unified the understanding of the micro-mechanism of LC failure of UD composites [10].

In the process of exploring the LC failure mode of UD composites, many scholars proposed different criteria, including macro-mechanical and meso-mechanical criteria. Early classical theories include the description of LC failure in UD composites in terms of maximum failure stresses proposed by Jenkins [11] and Stowell [12]. Tsai and Wu [13] proposed a failure criterion uniformly expressed in tensor form by considering the interactions among the stress components, which was later improved by Li and Chen et al. [14,15]. Subsequently, Puck [16] proposed a coupled compression–shear model, which introduces a correction factor based on the maximum stress criterion to more accurately describe the shear effect. Yen [17] considered the effect of transverse compressive stress on LC failure, and this model gave good results in analyzing the failure of composites in high-speed impacts, while Li et al. [18,19] analyzed the mechanism of fiber failure in low-speed impacts. In addition, Huang et al. [20] proposed a bridge-linking model using principal fiber meso-stresses to describe LC failure and later compared macro- and meso-mechanical strength theories, pointing out the advantages and prospects of the meso-mechanical strength theory. Davila et al. [21] first considered the fiber kinking pattern that occurs during LC failure and considered the improvement of the Hashin IFF criterion, and they then applied it to LC failure by establishing the LaRC02 criterion. Afterward, Pinho and Camanho et al. [22,23,24] constructed LaRC03~05 criteria through the consideration of the in situ effect, the nonlinear shear effect, and the influence of hydrostatic pressure, respectively. Further, Camanho [25] et al. applied a 3D kink model based on an invariant failure criterion to predict LC failure. In general, these aforementioned works were extensively applied to the strength design of composite structures with very far-reaching engineering value.

Experimental evidence [26,27] suggests that the formation of fiber kinking bands is the dominant failure mode during the LC of UD composites. The LaRC02 criterion applies the improved Hashin IFF criterion [28], which is derived from stress invariants, to the fiber kink failure mode. However, while theoretical considerations dictate that the tensile/compressive failure modes of the matrix should be distinguished by the sign of the normal stress $\sigma_{n}$ on the failure plane, the Hashin criterion employs a stress-invariant approach to formulate the matrix failure function, using the sign of $\sigma_{2}+\sigma_{3}$ to differentiate matrix failure modes. This methodology introduces inherent limitations. In contrast, Puck et al. developed their matrix failure criterion using stress components ($\sigma_{n}$, $\tau_{\mathrm{nt}}$, $\tau_{\mathrm{nl}}$) acting on the failure plane itself [28]. Therefore, under complex loading conditions and multiaxial stress states, selecting an IFF criterion based on the failure surface theory can provide more accurate failure predictions [29]. For this reason, three Puck-like matrix failure theories are selected in this work: the Puck criterion [16], Guo criterion [30], and Li criterion [31], which are in good agreement in determining the transverse failure of UD composites. These criteria exhibit dual advantages over the stress-invariant-based Hashin criterion implemented in LaRC02: the superior physical fidelity to failure mechanisms and consistently enhanced predictive accuracy in failure simulations. Their extension to compressive kinking configurations of fibers represents both an advancement of the LaRC criteria framework—providing novel methodologies for refining LaRC02-05 failure criteria—and a strategic complement to Puck-type criteria for fiber failure prediction. These IFF criteria are extended to LC failure determination of UD composites, and the rationality of the proposed criteria is verified using experimental data from the existing literature. The extension methodology adopted in this work helps to further improve the understanding of the LC failure mechanism and strength prediction, which has significant theoretical significance and engineering reference value.

The remaining work can be summarized as follows: Section 2 provides a discussion and assumptions on the LC failure mechanism of UD composites. Section 3 gives the derivation of the proposed criteria. Experimental validation and comparative analysis of the proposed criteria are given in Section 4. Section 5 discusses the influence of several material properties on the provided criteria under different stress states. Finally, Section 6 summarizes and prospects this work.

## 2. Mechanism of LC Failure in UD Composites

**Figure 1:**
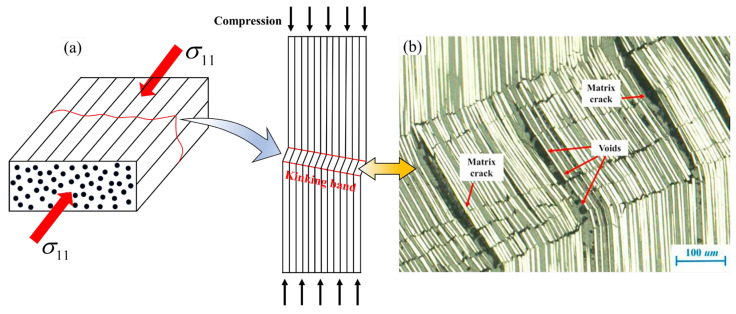
(**a**) Schematic and (**b**) actual micrographs of unidirectional composites subjected to LC failure (adapted with permission from [35], Elsevier, 2018).

UD composites exhibit complex mechanical behavior during the LC process, making it difficult for existing LC prediction models to evaluate them comprehensively and accurately. Gutkin et al. [32] conducted in situ observation experiments of the LC process in UD composites, using a one-sided notched compact compression specimen, intended to create stress concentration, with high-resolution cameras employed to capture and analyze the progressive stages of kink-band formation, propagation, and bandwidth broadening under the compressive loading of fibers, to investigate the physical mechanisms of LC failure intensively. The experimental results show that, depending on the difference in stress states, LC failure can be presented in three different modes: kinking (see Figure 1a), fiber shear, and micro-buckling [10].

So far, scholars do not have a unified understanding of the micro-mechanism of the LC process in UD composites. Puck et al. [33] concluded that a fiber failure mode occurs in UD composites when the stress in the fibers reaches the LC strength and proposed a series of criteria based on the failure surface theory. Argon et al. [34] concluded that there is an initial deflection angle in the fiber arrangement due to the fiber-reinforced composites containing tiny defects, resulting in LC making the deflected fibers rotate, and the fiber rotation increases with increasing compressive load. When compression reaches a certain level, the shear between the fibers leads to progressive damage to the matrix that supports the fibers, and the fibers subsequently fracture under the combined effect of shear and compression. Synthesizing the above research, Pinho [35] summarized that the occurrence of fiber kinking failure is due to the matrix damage caused by the shear between fibers under the action of significant LC, resulting from the initial misalignment angle of the fibers. When the matrix in the kinking band is damaged and can no longer support the fibers, the composites will experience LC failure, as shown in Figure 1a.

Actually, during the LC failure of composites, the two failure modes, fiber shear and micro-buckling, occur relatively rarely, with the folding formed by fiber kinking bands as the main failure mode. Accordingly, the LaRC02 criterion applies the Hashin IFF criterion, which has a relatively simple expression form, to the fiber kinking mode to establish an LC failure criterion for UD composites. However, the Hashin criterion is derived based on stress invariants rather than from physical mechanisms, leading to a certain lack of physical meaning of the LaRC02 criterion. In this work, based on this idea, several existing Puck-like criteria for IFF failure of UD composites are extended to LC failure determination, and a rationality assessment and parametric discussion of several criteria are performed.

## 3. Extension of Methods of IFF Criteria to LC Failure of UD Composites

### 3.1. Coordinate System Conversion

**Figure 2:**
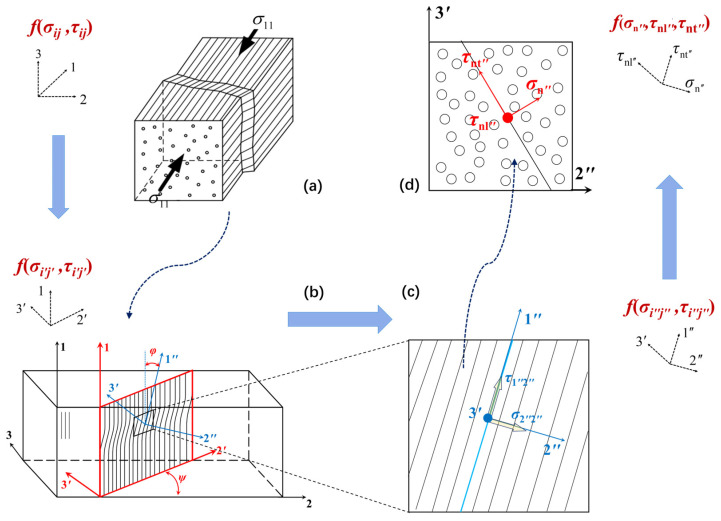
Schematic of LC coordinate system conversion for UD composites: (**a**) The transverse stress in the fiber kinking band (*σ_ij_*, *τ_ij_*) on the coordinate system(1, 2, 3), (**b**) (*σ_i’j’_*, *τ_i’j’_*) on the coordinate system (1’, 2’, 3’), (**c**) (*σ_i’’j’’_*, *τ_i’’j’’_*) on the coordinate system (1’, 2’, 3’) and (**d**) (*σ_n’’_, τ_nl’’_, τ_nt’’_*) on the coordinate system (1’’, 2’’, 3’’).

From the above, it is clear that the fiber kinking failure of UD composites is caused by the shear bending of the fibers due to the matrix damage, resulting in the formation of kinking bands. The mechanism of fiber kinking is similar to that of matrix failure. The main difference between them is that in the analysis of matrix failure, the stress is calculated based on the fracture surface, while in the analysis of fiber kinking failure, the stress is calculated based on the kinking plane. Additionally, the theory of shear bending can only be applicable to the compressive failure modes of fibers. Therefore, Pinho et al. [36] believe that the expression of the matrix failure criterion of composites can be used to construct the failure criterion under this LC mode, that is, the LC stress can be transformed into the transverse stress in the fiber kinking band through coordinate transformation. Here, the coordinate system (1, 2, 3) represents the material coordinate system, the coordinate system (1, 2′, 3′) represents the fiber kinking failure surface coordinate system, and the coordinate system (1″, 2″, 3′) represents the off-axis fiber kinking failure surface coordinate system. Thus, the stress components of the coordinate system (1″, 2″, 3′), that is, the transverse stress in the fiber kinking band, can be obtained, as shown in Figure 2.

(i) As shown in Figure 2a,b, the stress transformation from coordinate system $\left(1,\;2,\;3\right)$ to $\left(1,\;2^{\prime },\;3^{\prime }\right)$ is as follows:
  (1) $$\left\{\begin{array}{l} \sigma_{2^{\prime }2^{\prime }}=\frac{\sigma_{22}+\sigma_{33}}{2}+\frac{\sigma_{22}-\sigma_{33}}{2}\cos\left(2\psi \right)+\tau_{23}\sin\left(2\psi \right)\\ \sigma_{3^{\prime }3^{\prime }}=\sigma_{22}+\sigma_{33}-\sigma_{2^{\prime }2^{\prime }}\\ \tau_{12^{\prime }}=\tau_{12}\cos\psi +\tau_{31}\sin\psi \\ \tau_{2^{\prime }3^{\prime }}=0\\ \tau_{3^{\prime }1}=\tau_{31}\cos\psi -\tau_{12}\sin\psi \\ \psi =\frac{1}{2}\arctan\frac{2\tau_{23}}{\sigma_{22}-\sigma_{33}} \end{array}\right.$$
  where ψ is the angle between the kinking plane and the 2-axis.
(ii) As shown in Figure 2b,c, the stress transformation from coordinate system $\left(1,\;2^{\prime },\;3^{\prime }\right)$ to $\left(1^{\prime \prime },2^{\prime \prime },3^{\prime }\right)$ is as follows:
  (2) $$\left\{\begin{array}{l} \sigma_{1^{\prime \prime }1^{\prime \prime }}=\frac{\sigma_{11}+\sigma_{2^{\prime }2^{\prime }}}{2}+\frac{\sigma_{11}-\sigma_{2^{\prime }2^{\prime }}}{2}\cos\left(2\varphi \right)+\tau_{12^{\prime }}\sin\left(2\varphi \right)\\ \sigma_{2^{\prime \prime }2^{\prime \prime }}=\sigma_{11}+\sigma_{2^{\prime }2^{\prime }}-\sigma_{1^{\prime \prime }1^{\prime \prime }}\\ \tau_{1^{\prime \prime }2^{\prime \prime }}=-\frac{\sigma_{11}-\sigma_{2^{\prime }2^{\prime }}}{2}\sin\left(2\varphi \right)+\tau_{12^{\prime }}\cos\left(2\varphi \right)\\ \tau_{2^{\prime \prime }3^{\prime }}=\tau_{2^{\prime }3^{\prime }}\cos\varphi -\tau_{3^{\prime }1}\sin\varphi \\ \tau_{3^{\prime }1^{\prime \prime }}=\tau_{3^{\prime }1}\cos\varphi \end{array}\right.$$
  where $\varphi =\frac{\tau_{12^{\prime }}}{\left|\tau_{12^{\prime }}\right|}\left(\varphi_{0}+\varepsilon_{12^{\prime }}\right)$ is the deflection angle in the kinking plane, $\varphi_{0}$ is the initial deflection angle in the kinking plane, and $\varepsilon_{12^{\prime }}$ is the shear strain in the kinking plane, and they can be expressed as follows:
  (3) $$\left\{\begin{array}{l} \varphi_{0}=\left(1-\frac{X^{C}}{G_{12}}\right)\varphi^{C}\\ \varphi^{C}=\arctan\left(\frac{1-\sqrt{1-4\left(\frac{S_{12}}{X^{C}}+\mu_{12}\right)\left(\frac{S_{12}}{X^{C}}\right)}}{2\left(\frac{S_{12}}{X^{C}}+\mu_{12}\right)}\right)\\ \varepsilon_{12^{\prime }}=\frac{\varphi_{0}G_{12}+\left|\tau_{12^{\prime }}\right|}{G_{12}+\sigma_{11}-\sigma_{2^{\prime }2^{\prime }}}-\varphi_{0}\\ \mu_{12}=-\frac{S_{12}\cos\left(2\alpha_{0}\right)}{Y^{C}\cos^{2}\alpha_{0}} \end{array}\right.$$
  where $G_{12}$ is the longitudinal shear modulus of the composites and $\varphi^{C}$ is the misalignment angle under pure LC. Davila et al. [21] gave the calculation formula for the initial fiber deflection angle through theoretical derivation. $X^{C}$, $Y^{C}$, and $S_{12}$ are the LC strength, transverse compressive strength, and in-plane shear strength of the UD composites, respectively. $\mu_{12}$ is the longitudinal friction coefficient of the transverse failure surface. $\alpha_{0}$ is the friction angle, and for generalized fiber-reinforced resin-matrix composites, $\alpha_{0}\approx 53^{\circ }$.
(iii) As shown in Figure 2c,d, after obtaining each stress component in the coordinate system $\left(1^{\prime \prime },2^{\prime \prime },3^{\prime }\right)$, the stress component on the potential fracture surface of matrix cracking in the fiber kinking zone can be obtained. The stress component on the potential fracture surface is expressed in terms of the stress component in the coordinate system $\left(1^{\prime \prime },2^{\prime \prime },3^{\prime }\right)$ on the kinking surface of the fibers to obtain the expression for the stress component $\left({\sigma^{\prime \prime }}_{n}\left(\theta \right),{\tau^{\prime \prime }}_{nt}\left(\theta \right),{\tau^{\prime \prime }}_{nl}\left(\theta \right)\right)$:
  (4) $$\left\{\begin{array}{l} {\sigma^{\prime \prime }}_{n}\left(\theta \right)=\sigma_{2^{\prime \prime }2^{\prime \prime }}\cos^{2}\theta +\sigma_{3^{\prime }3^{\prime }}\sin^{2}\theta +2\tau_{2^{\prime \prime }3^{\prime }}\sin\theta \cos\theta \\ {\tau^{\prime \prime }}_{\mathrm{nt}}\left(\theta \right)=-\sigma_{2^{\prime \prime }2^{\prime \prime }}\sin\theta \cos\theta +\sigma_{3^{\prime }3^{\prime }}\sin\theta \cos\theta +\tau_{2^{\prime \prime }3^{\prime }}\left(\cos^{2}\theta -\sin^{2}\theta \right)\\ {\tau^{\prime \prime }}_{\mathrm{nl}}\left(\theta \right)=\tau_{3^{\prime }1^{\prime \prime }}\sin\theta +\tau_{1^{\prime \prime }2^{\prime \prime }}\cos\theta \end{array}\right.$$
  where *θ* is the potential fracture angle for matrix failure on the fiber kinking surface.

### 3.2. Application of Different IFF Criteria to LC Failure of UD Composites

In this work, several Puck-like physical-mechanism-based IFF criteria, including the Puck [16], Guo [30], and Li failure criteria [31], are selected to construct LC failure criteria for UD composites. Note that to distinguish them from the corresponding IFF criteria, the three criteria are hereafter denoted as LC-Puck, LC-Guo, and LC-Li failure criteria.

#### 3.2.1. LC-Puck Failure Criteria

For transverse tensile failure of UD composites (${\sigma^{\prime \prime }}_{n}\left(\theta \right)\geq 0$),

(5) $${\left(\frac{{\tau^{\prime \prime }}_{\mathrm{nt}}\left(\theta \right)}{R_{⊥⊥}^{A}}\right)}^{2}+{\left(\frac{{\tau^{\prime \prime }}_{\mathrm{nl}}\left(\theta \right)}{R_{⊥∥}^{A}}\right)}^{2}+\frac{2p_{⊥\Psi }^{T}{\sigma^{\prime \prime }}_{n}\left(\theta \right)}{R_{⊥\Psi }^{A}}+\left(1-\frac{2p_{⊥\Psi }^{T}R_{⊥}^{\mathrm{AT}}}{R_{⊥\Psi }^{A}}\right){\left(\frac{{\sigma^{\prime \prime }}_{n}\left(\theta \right)}{R_{⊥}^{\mathrm{AT}}}\right)}^{2}=1;$$

For transverse compressive failure of UD composites (${\sigma^{\prime \prime }}_{n}\left(\theta \right)<0$),

(6) $${\left(\frac{{\tau^{\prime \prime }}_{\mathrm{nt}}\left(\theta \right)}{R_{⊥⊥}^{A}}\right)}^{2}+{\left(\frac{{\tau^{\prime \prime }}_{\mathrm{nl}}\left(\theta \right)}{R_{⊥∥}^{A}}\right)}^{2}+\frac{2p_{⊥\Psi }^{C}{\sigma^{\prime \prime }}_{n}\left(\theta \right)}{R_{⊥\Psi }^{A}}=1$$

where

(7) $$\left\{\begin{array}{l} \frac{p_{⊥\Psi }^{T,C}}{R_{⊥\Psi }^{A}}=\frac{p_{⊥⊥}^{T,C}}{R_{⊥⊥}^{A}}\cos^{2}\Psi +\frac{p_{⊥∥}^{T,C}}{R_{⊥∥}^{A}}\sin^{2}\Psi \\ \cos^{2}\Psi =\frac{\tau_{\mathrm{nt}}^{2}}{\tau_{\mathrm{nt}}^{2}+\tau_{\mathrm{nl}}^{2}}\\ R_{⊥⊥}^{A}=\frac{Y^{C}}{2\left(1+p_{⊥⊥}^{c}\right)}\\ R_{⊥∥}^{A}=S_{12}\\ R_{⊥}^{\mathrm{AT}}=Y^{T} \end{array}\right.$$

where fracture resistance ($R_{⊥∥}^{A}$ or $R_{⊥⊥}^{A}$) is the ability of a stress (${\tau^{\prime \prime }}_{\mathrm{nt}}$ or ${\tau^{\prime \prime }}_{\mathrm{nl}}$) on an action surface to resist fracture of the material. $p_{⊥∥}^{T}$, $p_{⊥∥}^{C}$, $p_{⊥⊥}^{T}$, and $p_{⊥⊥}^{C}$ are the slope parameters of the contour lines of the failure envelope. In general, for-carbon fiber-reinforced composites, the slope parameter is in the range of 0.2~0.25; for glass-fiber-reinforced composites, the slope parameter is in the range of 0.25~0.3.

#### 3.2.2. LC-Guo Failure Criteria

For transverse tensile failure of UD composites (${\sigma^{\prime \prime }}_{n}\left(\theta \right)\geq 0$),

(8) $$\begin{array}{l} \left[\frac{1}{Y^{C}\sin^{2}\theta_{\mathrm{fp}}^{Y^{C}}+Y^{T}}\left(\frac{2Y^{C}\sin^{2}\theta_{\mathrm{fp}}^{Y^{C}}}{Y^{T}\cos^{2}\theta_{\mathrm{fp}}^{Y^{T}}}-\frac{Y^{T}}{Y^{C}\cos^{2}\theta_{\mathrm{fp}}^{Y^{C}}}\right)\right]{\sigma^{\prime \prime }}_{n}\left(\theta \right)+\\ \left[\frac{1}{Y^{C}\sin^{2}\theta_{\mathrm{fp}}^{Y^{C}}+Y^{T}}\left(\frac{2}{Y^{T}\cos^{2}\theta_{\mathrm{fp}}^{Y^{T}}}+\frac{1}{Y^{C}\cos^{2}\theta_{\mathrm{fp}}^{Y^{C}}}\right)-\frac{1}{{\left(Y^{T}\cos^{2}\theta_{\mathrm{fp}}^{Y^{T}}\right)}^{2}}\right]{\left({\sigma^{\prime \prime }}_{n}\left(\theta \right)\right)}^{2}+\\ \left[\frac{1}{Y^{C}\sin^{2}\theta_{\mathrm{fp}}^{Y^{C}}+Y^{T}}\left(\frac{2}{Y^{T}\cos^{2}\theta_{\mathrm{fp}}^{Y^{T}}}+\frac{1}{Y^{C}\cos^{2}\theta_{\mathrm{fp}}^{Y^{C}}}\right)\right]{\left({\tau^{\prime \prime }}_{\mathrm{nt}}\left(\theta \right)\right)}^{2}+\frac{{\left({\tau^{\prime \prime }}_{\mathrm{nl}}\left(\theta \right)\right)}^{2}}{{\left(S_{21}\cos\theta_{\mathrm{fp}}^{\mathrm{sl}}\right)}^{2}}=1 \end{array}$$

For transverse compressive failure of UD composites (${\sigma^{\prime \prime }}_{n}\left(\theta \right)<0$):

(9) $$\begin{array}{l} \left[\frac{1}{Y^{C}\sin^{2}\theta_{\mathrm{fp}}^{Y^{C}}+Y^{T}}\left(\frac{2Y^{C}\sin^{2}\theta_{\mathrm{fp}}^{Y^{C}}}{Y^{T}\cos^{2}\theta_{\mathrm{fp}}^{Y^{T}}}-\frac{Y^{T}}{Y^{C}\cos^{2}\theta_{\mathrm{fp}}^{Y^{C}}}\right)\right]{\sigma^{\prime \prime }}_{n}\left(\theta \right)+\\ \left[\frac{1}{Y^{C}\sin^{2}\theta_{\mathrm{fp}}^{Y^{C}}+Y^{T}}\left(\frac{2}{Y^{T}\cos^{2}\theta_{\mathrm{fp}}^{Y^{T}}}+\frac{1}{Y^{C}\cos^{2}\theta_{\mathrm{fp}}^{Y^{C}}}\right)\right]{\left({\tau^{\prime \prime }}_{\mathrm{nt}}\left(\theta \right)\right)}^{2}+\frac{{\left({\tau^{\prime \prime }}_{\mathrm{nl}}\left(\theta \right)\right)}^{2}}{{\left(S_{21}\cos\theta_{\mathrm{fp}}^{\mathrm{sl}}\right)}^{2}}=1 \end{array}$$

where $Y^{T}$, $Y^{C}$, and $S_{21}$ are the transverse tensile strength, transverse compressive strength, and in-plane shear strength of UD composites, respectively. $\theta_{\mathrm{fp}}^{Y^{T}}$, $\theta_{\mathrm{fp}}^{Y^{C}}$, and $\theta_{\mathrm{fp}}^{\mathrm{sl}}$ are the transverse tensile fracture angle, transverse compression fracture angle, and in-plane shear fracture angle of UD composites, respectively, for general resin-matrix composites. $\theta_{\mathrm{fp}}^{Y^{T}}\approx 0$, $\theta_{\mathrm{fp}}^{Y^{C}}\approx 53^{\circ }$, and $\theta_{\mathrm{fp}}^{\mathrm{sl}}\approx 0$.

#### 3.2.3. LC-Li Failure Criteria

For transverse tensile failure of UD composites (${\sigma^{\prime \prime }}_{n}\left(\theta \right)\geq 0$),

(10) $$\begin{array}{l} \frac{{\left({\tau^{\prime \prime }}_{\mathrm{nl}}\left(\theta \right)\right)}^{2}}{S_{21}^{2}}+\frac{{\left({\tau^{\prime \prime }}_{\mathrm{nt}}\left(\theta \right)\right)}^{2}}{{\left(Y^{C}\cos^{2}\theta_{\mathrm{fp}}^{Y^{C}}\right)}^{2}}+\frac{\sin^{2}\theta_{\mathrm{fp}}^{Y^{C}}-\cos^{2}\theta_{\mathrm{fp}}^{Y^{C}}}{Y^{C}\cos^{4}\theta_{\mathrm{fp}}^{Y^{C}}}{\sigma^{\prime \prime }}_{n}\left(\theta \right)+\\ {\left(\frac{1}{Y^{T}}\right)}^{2}\left[1-\frac{Y^{T}}{Y^{C}}\left(\frac{\sin^{2}\theta_{\mathrm{fp}}^{Y^{C}}-\cos^{2}\theta_{\mathrm{fp}}^{Y^{C}}}{\cos^{4}\theta_{\mathrm{fp}}^{Y^{C}}}\right)\right]{\left({\sigma^{\prime \prime }}_{n}\left(\theta \right)\right)}^{2}=1 \end{array}$$

For transverse compressive failure of UD composites (${\sigma^{\prime \prime }}_{n}\left(\theta \right)<0$),

(11) $$\frac{\sin^{2}\theta_{\mathrm{fp}}^{C}-\cos^{2}\theta_{\mathrm{fp}}^{C}}{Y^{C}\cos^{4}\theta_{\mathrm{fp}}^{C}}{\sigma^{\prime \prime }}_{n}\left(\theta \right)+\frac{{\tau^{\prime \prime }}_{\mathrm{nl}}\left(\theta \right)}{S_{21}^{2}}+\frac{{\tau^{\prime \prime }}_{\mathrm{nt}}\left(\theta \right)}{{\left(Y^{C}\cos^{2}\theta_{\mathrm{fp}}^{C}\right)}^{2}}=1$$

## 4. Validation and Assessment of Different Criteria

### 4.1. Fundamental Mechanical Parameters of Various Composites

For the three proposed LC failure criteria of UD composites, the experimental data of various composites in Table 1 are extracted from the existing literature for assessment and analyzed in comparison with the LaRC02 criteria.

### 4.2. Validation and Assessment

**Figure 3:**
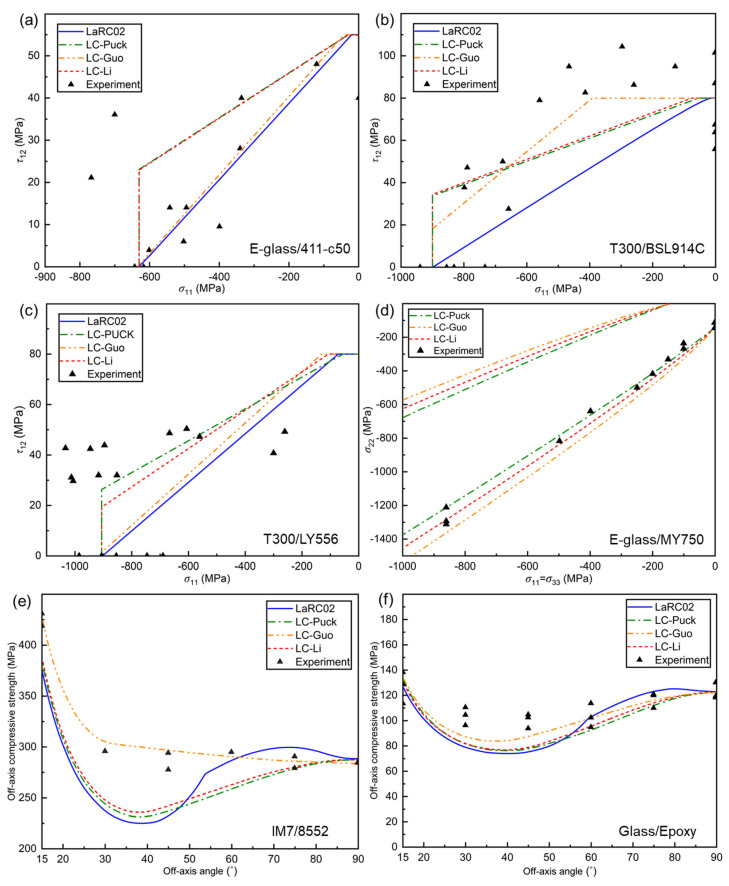
Comparison of failure envelopes of various composites under different stress states with experimental data: (**a**) E−glass/411−C50 [37], (**b**) T300/BSL914C [38], and (**c**) T300/LY556 [39] at (*σ*_11_, *τ*_12_); (**d**) E−glass/MY750 [40] at (*σ*_11_ = *σ*_33_, *σ*_22_); (**e**) IM7/8552 [25] and (**f**) Glass/Epoxy [41] under off-axial compression loading.

**Figure A1:**
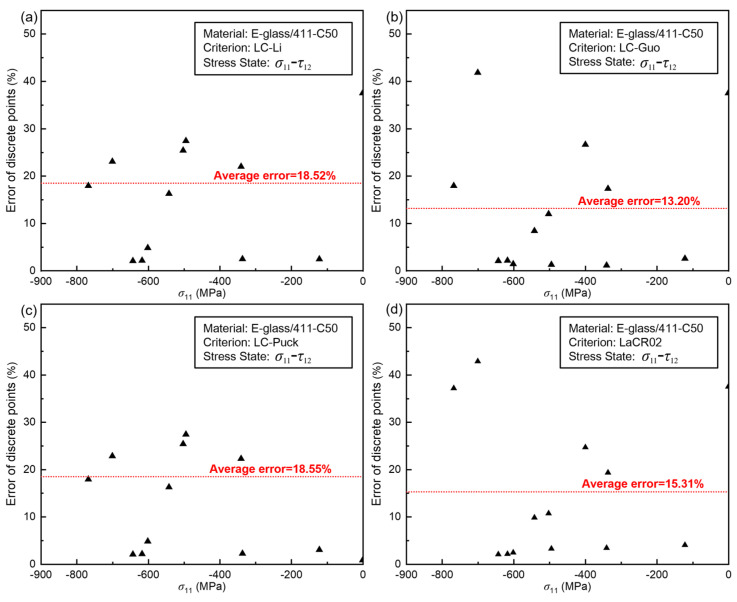
Experiment data and predicted results with different failure criteria for E−glass/411−C50 at (σ_11_, τ_12_). (**a**) LC−Li, (**b**) LC−Guo, (**c**) LC−Puck, and (**d**) LaCR02 failure criteria.

**Figure A2:**
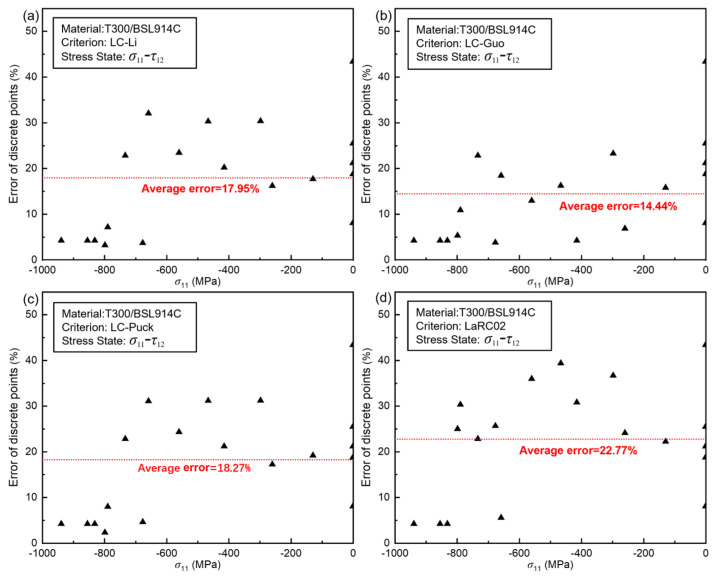
Experiment data and predicted results with different failure criteria for T300/BSL914C at (σ_11_, τ_12_). (**a**) LC−Li, (**b**) LC−Guo, (**c**) LC−Puck, and (**d**) LaCR02 failure criteria.

**Figure A3:**
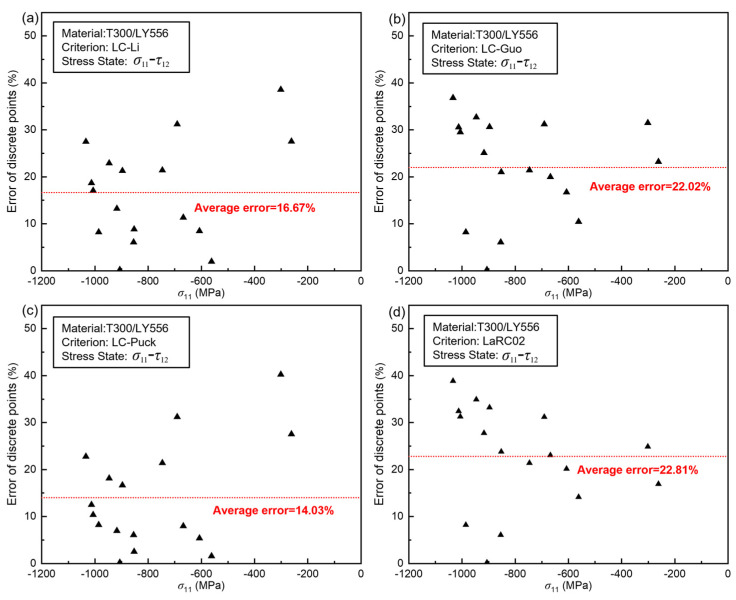
Experiment data and predicted results with different failure criteria for T300/LY556 at (σ_11_, τ_12_). (**a**) LC−Li, (**b**) LC−Guo, (**c**) LC−Puck, and (**d**) LaCR02 failure criteria.

**Figure A4:**
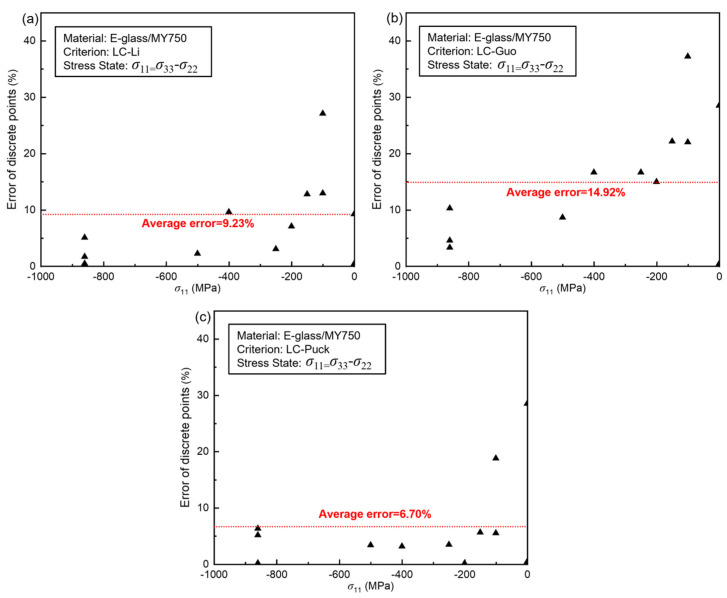
Experiment data and predicted results with different failure criteria for E−glass/MY750 at (σ_11_ = σ_33_, σ_22_). (**a**) LC−Li, (**b**) LC−Guo, and (**c**) LC−Puck failure criteria.

**Figure A5:**
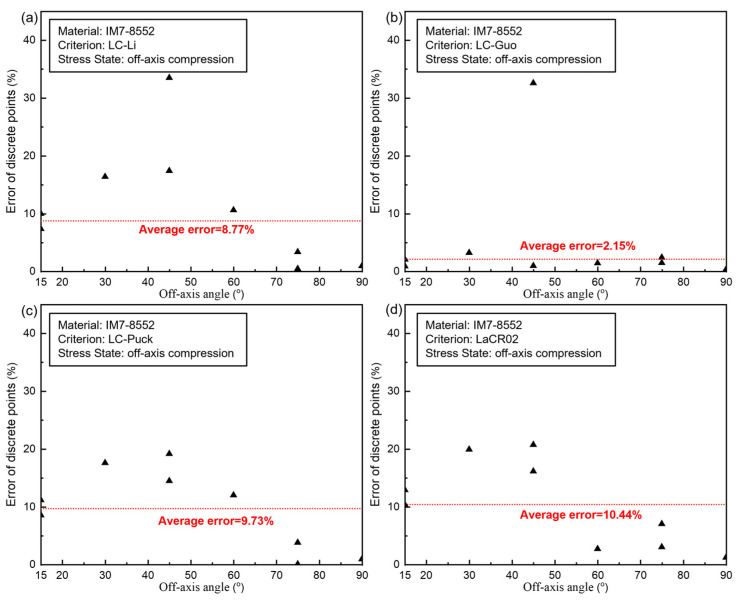
Experiment data and predicted results with different failure criteria for IM7−8552 under off−axial compression loading. (**a**) LC−Li, (**b**) LC−Guo, (**c**) LC−Puck, and (**d**) LaCR02 failure criteria.

**Figure A6:**
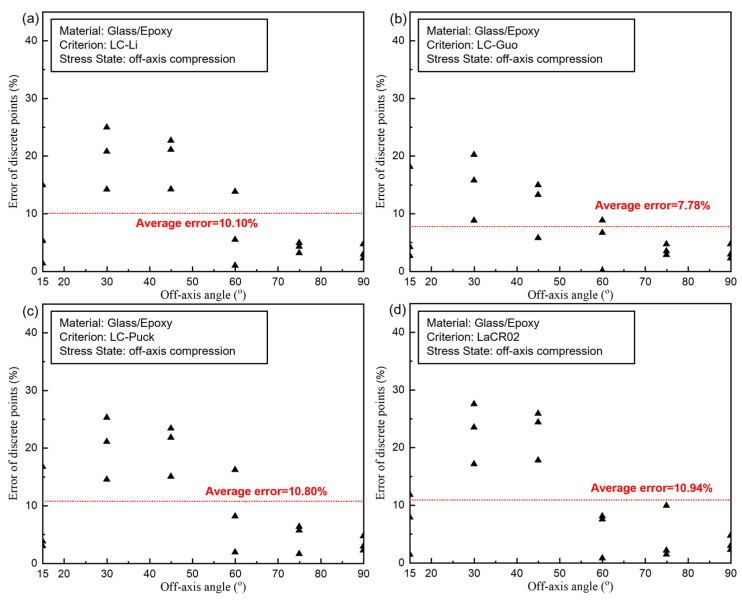
Experiment data and predicted results with different failure criteria for Glass/Epoxy under off−axial compression loading. (**a**) LC−Li, (**b**) LC−Guo, (**c**) LC−Puck, and (**d**) LaCR02 failure criteria.

Figure 3 displays the failure envelope and off-axial strength curves of various composites with different criteria and is compared with the experimental data. To analyze the prediction accuracy of different criteria more intuitively, Table 2 presents a comparison of mean errors predicted by different criteria for different stress states (the specific comparison errors can be found in Figure A1, Figure A2, Figure A3, Figure A4, Figure A5 and Figure A6 of Appendix A). In general, the results predicted by the three proposed criteria are within the engineering allowable range, and the prediction accuracy is slightly higher than that of the LaRC02 criterion, verifying the reasonableness of the three proposed criteria.

Figure 3a–c show the failure envelopes obtained by using the three proposed criteria and LaRC02 criterion for E-glass/411-C50, T300/BSL914C, and T300/LY556 under the $\left(\sigma_{11},\tau_{12}\right)$ stress state and are compared with the corresponding experimental data. It can be seen that the LaRC02 failure criterion predicts the most conservative results, followed by the LC-Puck and LC-Li failure criteria. However, LC-Guo has different degrees of opening in different materials. The denominator of the coefficient of the LC-Li and LC-Puck criterion terms is a positive $Y^{C}$ sign, and the denominator of the coefficient of the LC-Guo is a negative $Y^{C}$ sign and contains the $Y^{T}$ term, as analyzed in Section 5.3.2 and Section 5.3.3; as the $Y^{C}$ increases and the $Y^{T}$ decreases, the material’s intrinsic embrittlement increases; thus, the LC-Guo criterion is more sensitive to the changes in the material’s intrinsic embrittlement, so the openings are different in the stress state. Table 1 shows that the T300/BSL914C has the greatest intrinsic embrittlement, so LC-Guo predicts the greatest opening. Nevertheless, for E-glass/411-C50 and T300/LY556, the envelope curves of the three provided criteria are similar, while for T300/BSL914C, the envelope curve of the LC-Guo criterion is significantly more open than other criteria. In Table 2, it can be seen that for E-glass/411-C50, the LaRC02 failure criterion is more accurate than the three proposed criteria, with about 3% lower error. For T300/BSL914C and T300/LY556, the prediction accuracies of the three proposed criteria are significantly higher than those of the LaRC02 criterion, especially the LC-Guo criterion, with an error nearly 10% lower.

Figure 3d displays the failure envelope curves for E-glass/LY750 under the $\left(\sigma_{11}=\sigma_{33},\sigma_{22}\right)$ stress state using the three proposed criteria and is compared with the experimental data. Note that the LaRC02 criterion is not used for this stress state, mainly because it does not include $\sigma_{33}$. Each failure criterion assumes that the composites can withstand infinite stress in the hydrostatic compressive stress state [42,43]; thus, the predicted failure envelope has an open trend. From the images, it can be seen that the envelope curves of the three proposed criteria are similar, and all of them show excellent consistency with the experimental results. From Table 2, it can be seen that the envelope curves of the LC-Puck and LC-Li failure criteria are closer to the experimental data, and the prediction error of the LC-Guo failure criterion is marginally higher than that of the other two criteria. It is reasonable that the LC-Guo criterion demonstrates superior predictive accuracy for materials with high intrinsic brittleness, whereas its performance may be less precise than that of the LC-Li and LC-Puck criteria when applied to materials exhibiting lower intrinsic brittleness.

Figure 3e,f show the predicted off-axial compression strength curves for IM7/8552 and Glass/Epoxy with various criteria, respectively. For both materials, the LC-Guo failure criterion best matches the experimental results in the region of off-axis angles less than 55°, whereas the LC-Puck, LC-Li, and LaRC02 failure criteria predicted results with larger errors, and the former two are slightly more accurate than the LaRC02 criterion. In addition, the predicted curves of the four criteria show excellent consistency with the experimental results when the off-axial angle exceeds 55°. As can be seen from Table 2, the prediction accuracies of the three proposed criteria are higher than that of the LaRC02 criterion, and the LC-Guo failure criterion has the highest accuracy, with a minimum error of only 2.15%. It is worth noting that the off-axial compressive strength curves predicted by the LaRC02 criterion undergo a mode transition from shear to compressive fracture, and it is difficult to observe the transition point in the curves predicted by the other criteria.

## 5. Parameter Evaluations

Taking E-Glass/LY556 as a representative case, it is proposed to investigate the effects of the in-plane shear strength $S_{12}$, transverse compressive strength $Y^{C}$, and transverse tensile strength $Y^{T}$ of UD composites on the three proposed criteria under several typical stress states, i.e., $\left(\sigma_{11}=\sigma_{33},\;\sigma_{22}\right)$, $\left(\sigma_{11},\;\tau_{12}\right)$, and off-axial compression.

### 5.1. The Influence of φ_0_ on the Failure Envelope Curve

**Figure 4:**
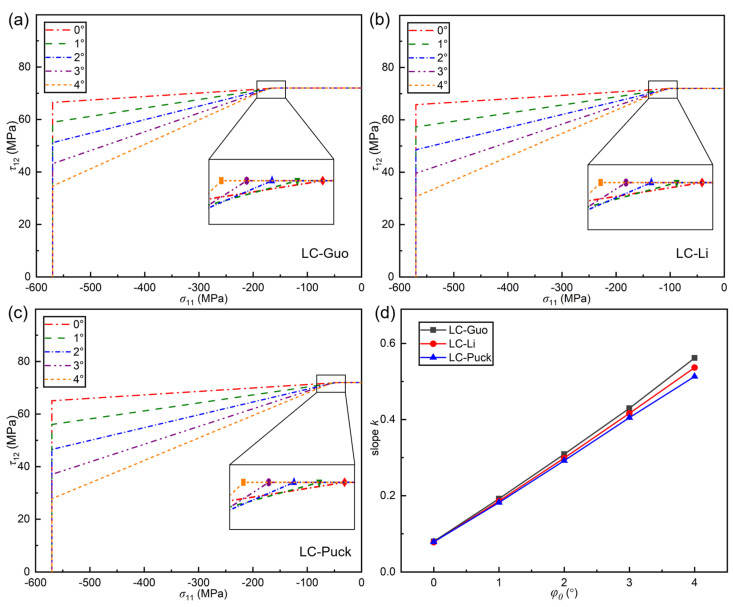
The influence of different $\varphi_{0}$ values on the failure envelope curve: (**a**) LC−Guo, (**b**) LC−Li, and (**c**) LC−Puck failure criteria; (**d**) the slope changes of the three envelope curves with different $\varphi_{0}$ values.

According to Argon’s [34] hypothesis, due to the presence of tiny defects in fiber-reinforced composites, there is a local initial misalignment angle in the fiber arrangement. Due to inherent micro-scale defects during manufacturing, fiber-reinforced composites exhibit localized initial misalignment angles in their fiber arrangements—an unavoidable production characteristic. While experimental measurement of these initial misalignment angles is prohibitively complex, Davila et al. derived a theoretical formulation to calculate the initial fiber misalignment angle, as presented in Equation (3). The physical meaning of $\varphi_{0}$ is to quantify the severity of the initial defects. $\varphi_{0}=0$ indicates that the material is intact and has no initial defects. As $\varphi_{0}$ increases, the severity of the initial defects increases. The influence of the initial fiber misalignment angle $\varphi_{0}$ on the failure envelope curves of the LC-Guo, LC-Li, and LC-Puck failure criteria under the (*σ*_11,_ *τ*_12_) stress state is analyzed, as shown in Figure 4. The slope of the envelope curve is *k*, and here we only quantify the change in the shape of the failure envelope curve.

It can be seen from Figure 4a–c that under the (*σ*_11,_ *τ*_12_) stress state, the failure envelope curves of the three criteria all have three parts. Specifically, for the horizontal part, the material failure is governed by in-plane shear; for the vertical part, the material failure is controlled by longitudinal compression; and for the inclined part, the material failure is jointly determined by in-plane shear and longitudinal compression. Obviously, with the increase in $\varphi_{0}$, the envelope curves of all three criteria change similarly, and their ranges all decrease. The horizontal part increases slightly, and the vertical part decreases. As can be observed from Figure 4d, the slope *k* of the inclined part increases with the increase in $\varphi_{0}$. In conclusion, using different initial deflection angles $\varphi_{0}$ in the calculation process has an impact on the shape and range of different failure envelopes.

### 5.2. Parametric Analysis of the (σ_11_ = σ_33,_ σ_22_) Stress State

#### 5.2.1. Effect of *S*_12_ on Failure Envelope Curves

**Figure 5:**
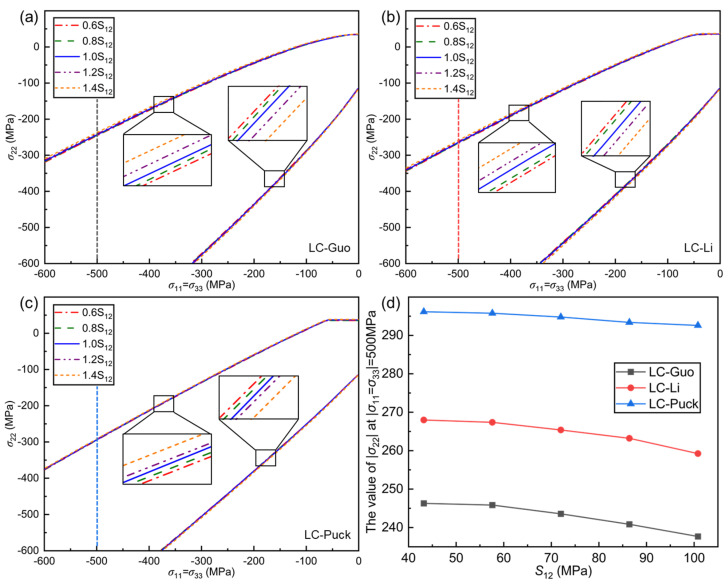
Effect of the in-plane shear strength *S*_12_ on the envelope curves for three criteria at (*σ*_11_ = *σ*_33_, *σ*_22_): (**a**) LC−Guo, (**b**) LC−Li, and (**c**) LC−Puck failure criteria; (**d**) relationship between the value of |*σ*_22_| at |*σ*_11_ = *σ*_33_| = 500 MPa in different envelope curves and *S*_12_.

The effect of in-plane shear strength $S_{12}$ is analyzed on the failure envelope curves of the LC-Guo, LC-Li, and LC-Puck failure criteria under the $\left(\sigma_{11}=\sigma_{33},\;\sigma_{22}\right)$ stress state, as shown in Figure 5.

From Figure 5a–c, it can be seen that the larger the in-plane shear strength $S_{12}$ is in the $\left(\sigma_{11}=\sigma_{33},\;\sigma_{22}\right)$ stress state, the larger the opening of the envelope curves of the proposed three criteria is, and the predictions of the criteria become more conservative, but the effect is negligible in general. $\left|\sigma_{22}\right|$ at $\sigma_{11}=\sigma_{33}=-500\;\mathrm{MPa}$ is selected for analysis as shown in Figure 5d. It can be seen that when $S_{12}$ is enlarged from 0.6 to 1.4 times, $\left|\sigma_{22}\right|$ decreases by only about 3%.

From the expressions of the three proposed criteria, it can be seen that the term containing $S_{12}$ is independent of the other terms, thus leading to a smaller change in the envelope. Moreover, the maximum value of ${\tau^{\prime \prime }}_{\mathrm{nl}}$ is increased when $S_{12}$ is increased, while ${\tau^{\prime \prime }}_{\mathrm{nl}}$ is calculated to be related to $\sigma_{11}$ and $\sigma_{22}$; when $\sigma_{11}$ is constant, it corresponds to a decrease in $\left|\sigma_{22}\right|$, and the stress at failure decreases. It is generally believed that UD composites exhibit isotropy in the plane perpendicular to the fibers (transversely isotropic). Therefore, the plasticity degree of the composite material can be defined by the ratio of compressive and tensile strength [43]. From the properties of the material, the increase in $S_{12}$ does not modify the material’s intrinsic brittleness, further explaining the minor change in the envelope. However, non-equivalent three-direction compression causes a change in the shape of the material, which is directly related to $S_{12}$; i.e., the larger $S_{12}$ is, the larger the absolute value of the maximum stress at failure is.

#### 5.2.2. Effect of *Y*^C^ on Failure Envelope Curves

**Figure 6:**
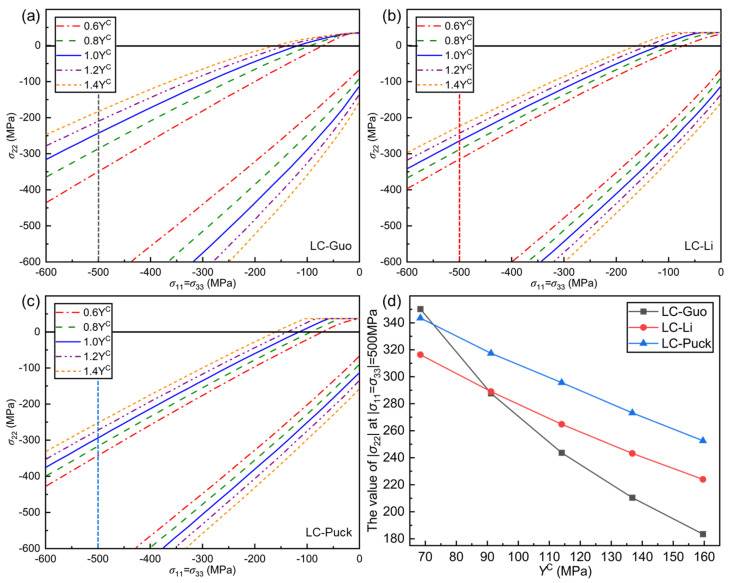
Influence of *Y*^C^ on the envelope curves for three criteria at (*σ*_11_ = *σ*_33_, *σ*_22_): (**a**) LC−Guo, (**b**) LC−Li, and (**c**) LC−Puck failure criteria; (**d**) relationship between the value of |*σ*_22_| at |*σ*_11_ = *σ*_33_| = 500 MPa in different envelope curves and *Y*^C^.

The influence of $Y^{C}$ is analyzed on the failure envelope curves of LC-Guo, LC-Li, and LC-Puck failure criteria under the $\left(\sigma_{11}=\sigma_{33},\;\sigma_{22}\right)$ stress state as shown in Figure 6. Compared to $S_{12}$, $Y^{C}$ has a more significant effect on the envelopes of the three proposed guidelines, which shows overall that the larger $Y^{C}$ is, the greater the expansion of the failure envelope curve is and the more conservative the predictions of the criteria are. From the expressions of the three proposed criteria, it can be seen that $Y^{C}$ is directly related to $\sigma_{11}$, $\sigma_{22}$, and $\sigma_{33}$, and an increase in $Y^{C}$ can significantly increase the opening of the failure envelope, which also leads to a significant decrease in the value of $\left|\sigma_{22}\right|$ at $\sigma_{11}=\sigma_{33}$. From the perspective of material properties, an increase in $Y^{C}$ and a steady $Y^{T}$ increase the intrinsic brittleness of the material, causing a more significant stress concentration effect during the loading process, which makes failure more likely.

As shown in Figure 6a–c, when $\sigma_{22}\geq 0$ and $\sigma_{11}=\sigma_{33}=0$, the envelopes of the LC-Guo failure criterion are smoothly converging to $Y^{T}$, while the other two criteria are forcibly converging to $Y^{T}$, with significant inflection points on the envelopes of these two criteria. This is due to the fact that ${\left(1/Y^{T}\right)}^{2}$ can be extracted independently from the coefficients of ${\left({\sigma^{\prime \prime }}_{n}\left(\theta \right)\right)}^{2}$ in the transverse tensile failure expressions of the LC-Li and LC-Puck criteria, while ${\left(1/Y^{T}\right)}^{2}$ cannot be extracted independently from the coefficients of the LC-Guo criterion, whose coefficients are coupled to each other.

When $\sigma_{22}<0$, the variation between the envelope curves is nonlinear for the LC-Guo failure criterion and linear for the other two criteria. This is because the coefficients of ${\sigma^{\prime \prime }}_{n}$ in the LC-Guo criterion contain the squared term of $Y^{C}$, whereas the other two criteria have the primary term of $Y^{C}$. The $\left|\sigma_{22}\right|$ at $\sigma_{11}=\sigma_{33}=-500\;\mathrm{MPa}$ is selected for analysis as shown in Figure 6d. It can be seen that when $Y^{C}$ increases from 0.6 to 1.4 times, $\left|\sigma_{22}\right|$ decreases by nearly 30% for the LC-Li and LC-Puck failure criteria, while $\left|\sigma_{22}\right|$ decreases by nearly 50% for the LC-Guo criterion; i.e., the change magnitude in the LC-Guo failure criterion is significantly larger than that in the other two criteria.

#### 5.2.3. Effect of *Y*^T^ on Failure Envelope Curves

**Figure 7:**
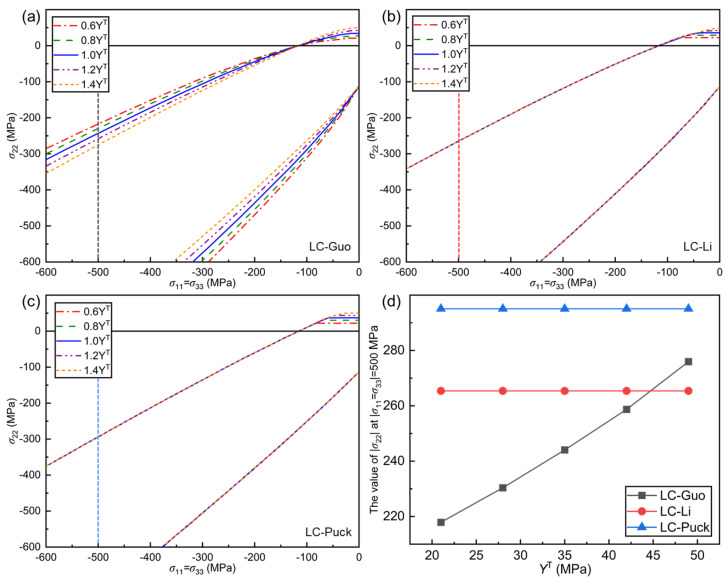
Influence of *Y*^T^ on the envelope curves for three criteria at (*σ*_11_ = *σ*_33_, *σ*_22_): (**a**) LC−Guo, (**b**) LC−Li, and (**c**) LC−Puck failure criteria; (**d**) relationship between the value of |*σ*_22_| at |*σ*_11_ = *σ*_33_| = 500 MPa in different envelope curves and *Y*^T^.

The influence of $Y^{T}$ is analyzed on the failure envelope curves of LC-Guo, LC-Li, and LC-Puck failure criteria under the $\left(\sigma_{11}=\sigma_{33},\;\sigma_{22}\right)$ stress state as shown in Figure 7. Compared to $Y^{C}$, the effect of $Y^{T}$ on the envelopes of the three proposed criteria is not very significant, and the overall performance is that the larger $Y^{T}$ is, the smaller the opening of the envelope curve of the LC-Guo criterion is, and the size of the opening of the envelope curve of the other two criteria remains unchanged. From the perspective of material properties, $Y^{C}$ remains steady, $Y^{T}$ becomes larger, the intrinsic brittleness of the material decreases, and the opening of the failure envelope curve should narrow.

As shown in Figure 7a–c, when $\sigma_{22}\geq 0$, the smaller $Y^{T}$ is, the smaller the failure envelope curve is, and the more conservative the prediction results of the criteria are. In addition, when $\sigma_{11}=\sigma_{33}=0$, the curve of the LC-Guo criterion is smoothly converging to $Y^{T}$, while the other two criteria are forcibly converging to $Y^{T}$ with inflection points on the curve, which is similar to the effect of $Y^{C}$ on the envelope curve.

When $\sigma_{22}<0$, the opening of the envelope curve of the LC-Guo failure criterion decreases with increasing $Y^{T}$, the more open the criterion predictions are, while $Y^{T}$ has almost no effect on the LC-Li and LC-Puck failure criteria. The reason for this is that when $\sigma_{22}<0$, the transverse compression failure expressions for the LC-Li and LC-Puck failure criteria do not have a $Y^{T}$ term, whereas the transverse compression failure expression for the LC-Guo failure criterion contains this term. The $\left|\sigma_{22}\right|$ at $\sigma_{11}=\sigma_{33}=-500\;\mathrm{MPa}$ is selected for analysis as shown in Figure 7d. It can be seen that when $Y^{T}$ is increased from 0.6 to 1.4 times, $\left|\sigma_{22}\right|$ for the LC-Li and LC-Puck failure criteria remains unchanged, while that for the LC-Guo failure criterion is improved by 27%.

### 5.3. Parametric Analysis of the (σ_11_, τ_12_) Stress State

#### 5.3.1. Effect of *S*_12_ on Failure Envelope Curves

**Figure 8:**
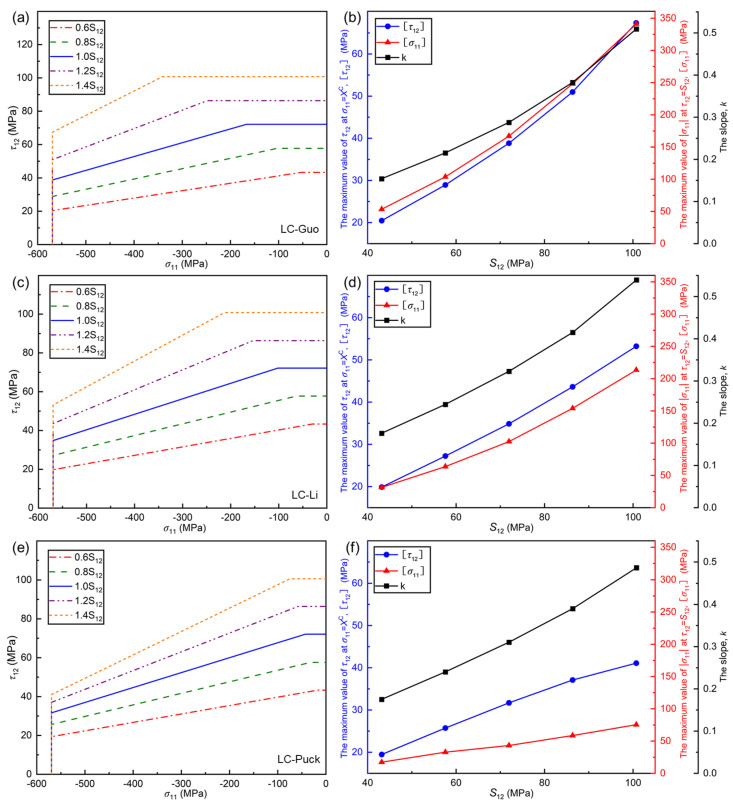
Influence of *S*_12_ on the envelope curves for three criteria at (*σ*_11_, *τ*_12_): (**a**) LC−Guo, (**c**) LC−Li, and (**e**) LC−Puck failure criteria, and relationships between some parameters in different envelope curves and *S*_12_: (**b**) LC−Guo, (**d**) LC−Li, and (**f**) LC−Puck failure criteria.

The effect of in-plane shear strength $S_{12}$ is analyzed on the failure envelope curves of LC-Guo, LC-Li, and LC-Puck failure criteria under the $\left(\sigma_{11},\;\tau_{12}\right)$ stress state as shown in Figure 8. Note that the maximum value of $\tau_{12}$ corresponding to $\sigma_{11}=X^{C}$ is defined as $\left[\tau_{12}\right]$; the maximum value of $\left|\sigma_{11}\right|$ corresponding to $\tau_{12}=S_{12}$ is defined as $\left[\sigma_{11}\right]$; and the slope of the failure envelope curve in the region is defined as *k*, which represents the influence of shear on the compressive strength of UD composites at failure.

These three aspects can be regarded as similar to those in Section 5.1. It is clear that the range of envelope curves for all three proposed criteria increases with the growth of $S_{12}$, where both the horizontal and vertical parts increase and the inclined part decreases. The envelope curve of the LC-Guo failure criterion changes the fastest, and the envelope curve of the LC-Puck failure criterion changes the slowest.

In Figure 8b,d,f, it can be seen that $\left[\tau_{12}\right]$, $\left[\sigma_{11}\right]$, and *k* for all three criteria increase with the growth of $S_{12}$, with only differences in the variation range and magnitude. Therein, the LC-Guo failure criterion has the largest variation range and magnitude of these three values, and the LC-Puck failure criterion has the smallest variation range and magnitude. From the LC failure mechanism of UD composites, it can be seen that although the increasing $S_{12}$ cannot modify the material’s intrinsic brittleness, the increase in $S_{12}$ significantly improves the inter-fiber shear-carrying capacity, which would result in the LC failure of UD composites being controlled to a large extent by $\tau_{12}$ and almost unaffected by $\sigma_{11}$.

#### 5.3.2. Effect of *Y*^C^ on Failure Envelope Curves

**Figure 9:**
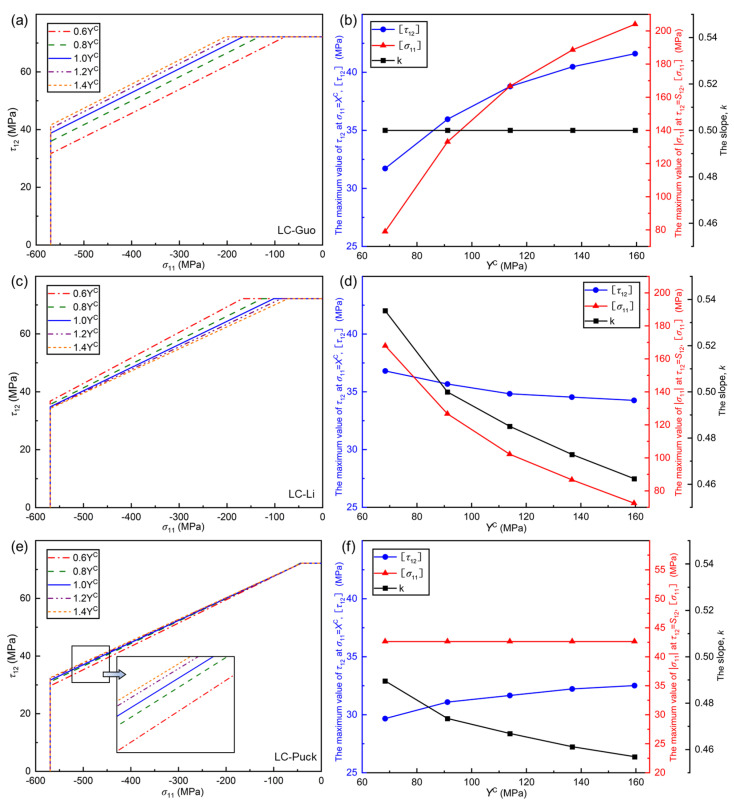
Influence of *Y*^C^ on the envelope curves for three criteria at (*σ*_11_, *τ*_12_): (**a**) LC−Guo, (**c**) LC−Li, and (**e**) LC−Puck failure criteria, and relationships between some parameters in different envelope curves and *Y*^C^: (**b**) LC−Guo, (**d**) LC−Li, and (**f**) LC−Puck failure criteria.

The influence of $Y^{C}$ is analyzed on the failure envelope curves of LC-Guo, LC-Li, and LC-Puck failure criteria under the $\left(\sigma_{11},\;\tau_{12}\right)$ stress state as shown in Figure 9. Similar to the above, three parameters are also defined, i.e., $\left[\tau_{12}\right]$, $\left[\sigma_{11}\right]$, and *k*.

From Figure 9a,c,e, it can be seen that the range of the envelope curves for the LC-Li and LC-Puck failure criteria show a decreasing trend with increasing $Y^{C}$, while the range of the envelope curves for the LC-Guo failure criterion shows an increasing trend. Therein, the envelope curve of the LC-Guo failure criterion has the largest variation, and the envelope curve of the LC-Puck failure criterion has the smallest variation. From the perspective of the criteria expression, the LC-Li and LC-Puck failure criteria have a positive sign with $Y^{C}$ in the denominator among the coefficients of the ${\sigma^{\prime \prime }}_{n}\left(\theta \right)$ item, while the LC-Guo failure criteria have a negative sign with $Y^{C}$ in the denominator among the coefficients of the ${\sigma^{\prime \prime }}_{n}\left(\theta \right)$ item, thus causing the above variation rule.

In Figure 9b,d,f, it can be seen that $\left[\tau_{12}\right]$ and $\left[\sigma_{11}\right]$ of the LC-Guo failure criterion increase with $Y^{C}$ increasing, and *k* is constant; $\left[\tau_{12}\right]$, $\left[\sigma_{11}\right]$, and *k* of the LC-Li failure criterion decrease with $Y^{C}$ increasing; and in the LC-Puck failure criterion, as $Y^{C}$ increases, $\left[\tau_{12}\right]$ increases, *k* decreases, and $\left[\sigma_{11}\right]$ remains constant. In terms of the failure mechanism, $Y^{C}$ increases and $Y^{T}$ remains constant, the material’s intrinsic brittleness increases, and transverse compression has a reinforcing effect on the in-plane shear. When Puck et al. [33] studied the transverse compression failure of intrinsically brittle materials, it was confirmed that the more significant the intrinsic brittleness is, the more obvious the strengthening effect is. Therefore, $\left[\tau_{12}\right]$, $\left[\sigma_{11}\right]$, and *k* should all show an increasing trend.

#### 5.3.3. Effect of *Y*^T^ on Failure Envelope Curves

**Figure 10:**
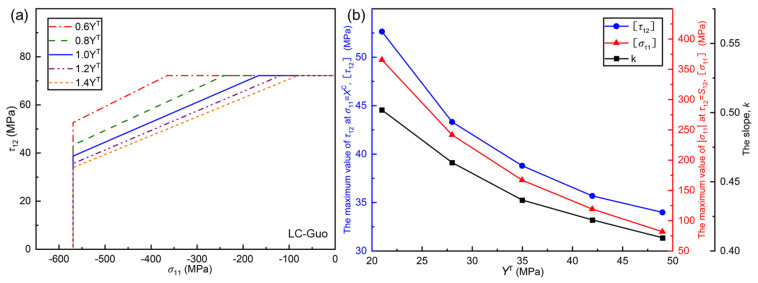
(**a**) Influence of *Y*^T^ on the envelope curves of LC−Guo criteria at (σ_11_, τ_12_); (**b**) relationships between some parameters in different envelope curves and *Y*^T^.

Figure 10 shows the effect of transverse tensile strength $Y^{T}$ on the envelope curve of the LC-Guo failure criterion. From the criteria expressions, it can be seen that the LC-Li and LC-Puck failure criteria do not contain a $Y^{T}$ term in the matrix compression failure, so $Y^{T}$ has no effect on the failure envelopes of these two criteria, which are not discussed here.

From Figure 10a, it can be seen that with the increase in $Y^{T}$, the range of the envelope curve of LC-Guo failure criterion reduces, and the prediction becomes conservative. Similarly, the trends of $\left[\tau_{12}\right]$, $\left[\sigma_{11}\right]$, and *k* with $Y^{T}$ are extracted, as shown in Figure 10b. It can be seen that all three parameters decrease with increasing $Y^{T}$. From the expression of the LC-Guo failure criterion, it can be seen that the $Y^{T}$ term comes after the denominator or negative sign and is quadratic; thus, the predicted failure envelope becomes more conservative, and the slope *k* decreases as $Y^{T}$ increases. In terms of material properties, $Y^{T}$ increases, $Y^{C}$ remains steady, the intrinsic brittleness of the material decreases, the effect of shear or compressive loading of the material alone in the $\left(\sigma_{11},\;\tau_{12}\right)$ state decreases, the combined effect of the multiaxial loading is enhanced, and the tilted portion of the envelope increases, thus leading to the fact that both $\left[\tau_{12}\right]$ and $\left[\sigma_{11}\right]$ decrease with $Y^{T}$ increasing.

### 5.4. Effect of Mechanical Parameters on Off-Axial Strength Prediction

#### 5.4.1. Effect of *S*_12_ on Off-Axial Compression Strength

**Figure 11:**
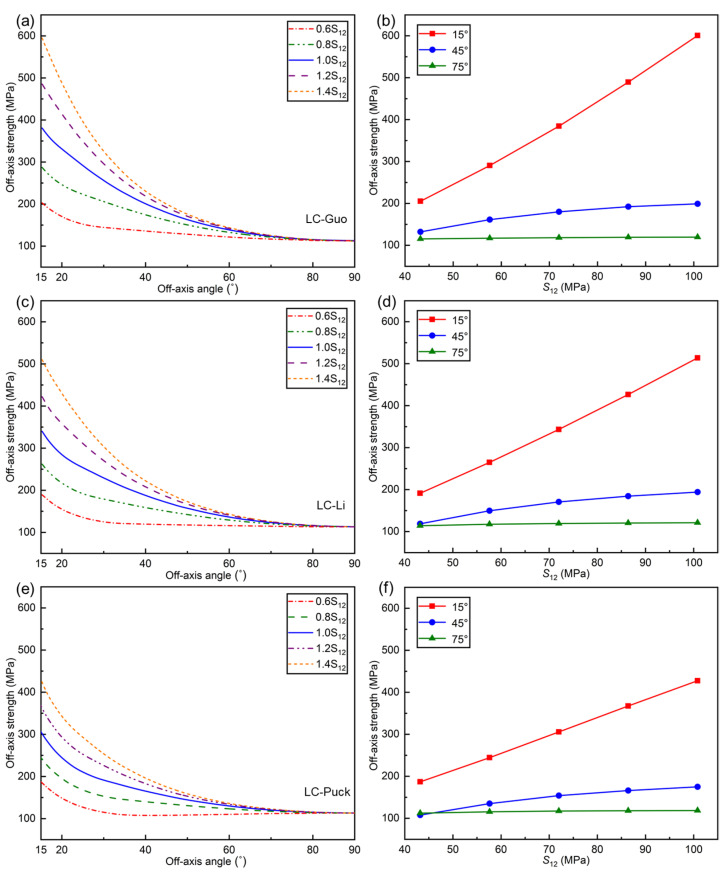
Influence of *S*_12_ on the off−axial compressive strength curves for three criteria: (**a**) LC−Guo, (**c**) LC−Li, and (**e**) LC−Puck failure criteria; tendency of off−axial compressive strength with S12 at determined angles: (**b**) LC−Guo, (**d**) LC−Li, and (**f**) LC−Puck failure criteria.

Figure 11 demonstrates the influence of $S_{12}$ on the off-axial compressive strength predicted by the LC-Guo, LC-Li, and LC-Puck failure criteria. In general, the effect of $S_{12}$ on the off-axial compressive strength predicted by these three criteria is similar.

From Figure 11a,c,e, it can be observed that the off-axial compressive strength predicted by all three criteria increases as $S_{12}$ increases. From the perspective of failure mechanism, the off-axial compressive damage in UD composites is fundamentally induced by shear stress, and the increase in shear strength inevitably improves the off-axial compressive strength of the material. An analysis of the three proposed criterion expressions shows that $S_{12}$ has no dependence on other parameters, so the predicted off-axial compressive strength necessarily increases when $S_{12}$ increases.

Furthermore, it can be observed from Figure 11b,d,f that when the off-axis angle is small, the off-axial compression strength improves with the increase in $S_{12}$, and the growth rate decreases with the increase in the off-axis angle. This occurs because the off-axial compression strength converges to LC strength $X^{C}$ at small off-axis angles, while the off-axial compression strength converges to the transverse compressive strength $Y^{C}$ when the off-axis angle is large. However, the off-axial compression strength exhibits significantly faster convergence to the LC strength of UD composites compared to its convergence toward transverse compressive strength, thereby mechanistically explaining the observed phenomenon.

#### 5.4.2. Effect of *Y*^C^ on Off-Axial Compression Strength

**Figure 12:**
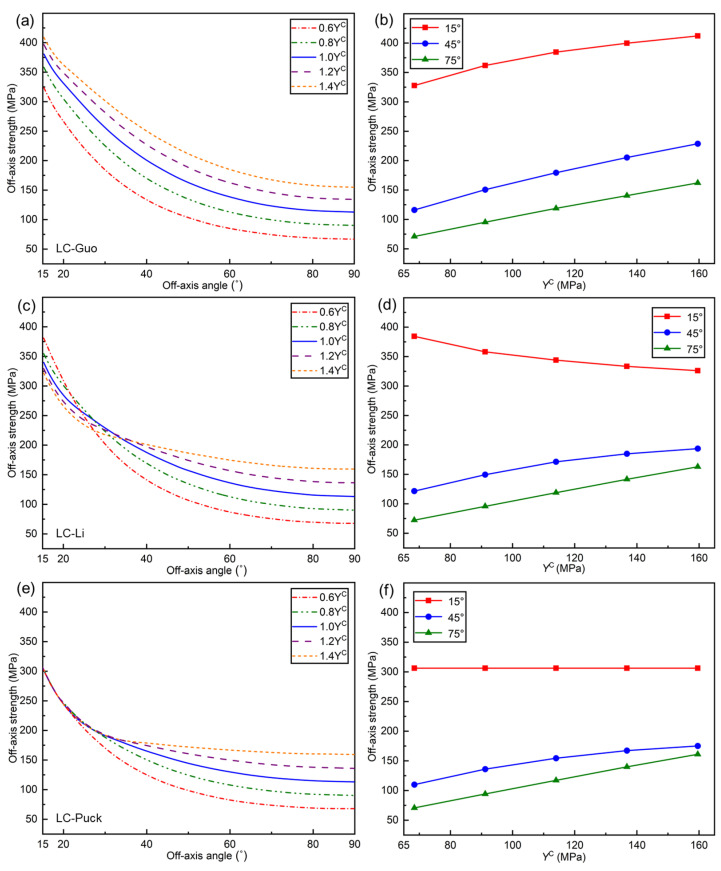
Influence of *Y*^C^ on the off−axial compressive strength curves for three criteria: (**a**) LC−Guo, (**c**) LC−Li, and (**e**) LC−Puck failure criteria; tendency of off−axial compressive strength with *Y*^C^ at determined angles: (**b**) LC−Guo, (**d**) LC−Li, and (**f**) LC−Puck failure criteria.

Figure 12 illustrates the influence of $Y^{C}$ on the off-axial compressive strength predicted by the LC-Guo, LC-Li, and LC-Puck failure criteria. From Figure 12a,c,e, it can be observed that when the off-axial angle exceeds 30°, the effect on the off-axial compression strength of the three proposed criteria is similar; i.e., the off-axial compression strength increases with the increase in $Y^{C}$, which is due to the fact that the off-axial compression strength curves converge to $Y^{C}$ as the off-axis angle increases.

However, there is a difference in the effect of $Y^{C}$ on the off-axial compression strength of the three criteria when the off-axis angle is less than 30°. Specifically, for the LC-Guo failure criterion, the off-axial compressive strength follows the same pattern as the effect of $Y^{C}$ on the off-axial compression strength (see Figure 12b). For the LC-Li failure criterion, the influence pattern on off-axial compression strength shifted between 20° and 30°, resulting in a reduction in off-axial compressive strength with increasing $Y^{C}$ (see Figure 12d). For the LC-Puck failure criterion, the off-axial compression strength curve gradually converges to a single curve when the off-axis angle is lower than 30°, and the effect of $Y^{C}$ on the off-axial compression strength diminishes (see Figure 12f).

From a mathematical point of view, the reasons for these differences are mainly due to differences in the expressions of the various criteria. In terms of material properties, $Y^{C}$ becomes larger and $Y^{T}$ remains unchanged, the material’s intrinsic brittleness is increased, and the strengthening effect of transverse compression on the in-plane shear is increased, thus leading to an increase in the off-axial compression strength. However, there is uncertainty in this law as the off-axis angle decreases, but it eventually converges to the LC strength $X^{C}$.

#### 5.4.3. Effect of *Y*^T^ on Off-Axial Compression Strength

**Figure 13:**
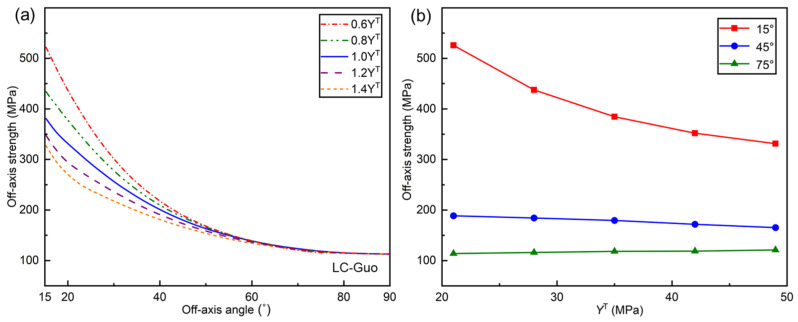
(**a**) Influence of *Y*^T^ on the off−axial compressive strength curves of LC−Guo criteria; (**b**) tendency of off−axial compressive strength with *Y*^T^ at determined angles.

Figure 13 displays the influence of $Y^{T}$ on the off-axial compression strength predicted by the LC-Guo failure criterion. From the criterion expressions, it can be seen that the LC-Li and LC-Puck failure criteria do not contain a $Y^{T}$ term in the matrix compression failure mode, so $Y^{T}$ has no effect on the off-axial compression strengths predicted by these two criteria, which are not discussed here.

From Figure 13a,b, it can be observed that the predicted off-axial compression strength decreases with the increase in $Y^{T}$. The reason is that the increase in $Y^{T}$ causes a reduction in the material’s inherent brittleness, which decreases the strengthening influence of transverse compression on in-plane shear, while the off-axial compression failure of UD composites is a shear failure in most cases and thus leads to the reduction in the off-axial compressive strength with the increase in $Y^{T}$.

## 6. Conclusions

In this work, several Puck-like physical-mechanism-based IFF failure criteria of UD composites are developed for evaluating their LC failure. Considering the most likely fiber kinking failure mode of UD composites in the LC process, a three-level coordinate system transformation is adopted to gradually transform the externally applied stress into the fiber kinking coordinate system, and several types of Puck-like failure criteria are introduced into this coordinate system to determine matrix cracking; thus, several criteria for evaluating the LC failure of UD composites are developed (denoted as LC-Guo, LC-Li, and LC-Puck failure criteria). On this basis, the influence of several typical material parameters on the proposed criteria is evaluated, and the following two important conclusions are obtained:

(1) Compared to the LaRC02 criterion, the proposed three criteria are slightly higher in overall prediction accuracy, especially the LC-Guo failure criterion, meaning that the proposed three criteria are reasonable. However, since factors such as in situ effects and matrix nonlinear shear are not considered, the overall error is still larger, averaging around 15%, and it could be further reduced in line with the development patterns of the LaRC02~05 criteria in the future.
(2) As $Y^{C}$ increases or $Y^{T}$ decreases, the intrinsic brittleness of UD composites increases, the failure envelope curves predicted by the three proposed criteria under different stress states tend to be conservative, and the predicted off-axial compression strength decreases in general. Additionally, the variation in $S_{12}$ does not cause a change in the intrinsic brittleness of the material, but it can improve the shear resistance of the material during the LC failure process, and its influence law on the mechanical properties of the material is basically similar to that of $Y^{C}$.

## Figures and Tables

**Table 1:** Fundamental mechanical parameters of several composites.

Material	*Y*^T^ (MPa)	*Y*^C^ (MPa)	*S*_21_ (MPa)	*X*^C^ (MPa)	*G*_12_ (MPa)
E-glass/411-C50 [1]	83.73	234.44	55	630	3650
T300/BSL914C [2]	27	200	80	900	5500
T300/LY556 [5]	50.02	140.06	80	905	2656
E-glass/MY750 [7]	40	145	73	800	5830
IM7/8552 [9]	73	185	90	1590	5600
Glass/Epoxy [10]	40.7	122.7	30.5	650	3400
E-glass/LY556 [11]	35	114	72	570	5830

**Table 2:** Average errors of various failure criteria.

Material	Stress State	LaRC02	LC-Puck	LC-Guo	LC-Li
E-glass/411-C50	$\left(\sigma_{11},\;\tau_{12}\right)$	15.31%	18.55%	13.20%	18.52%
T300/BSL914C	$\left(\sigma_{11},\;\tau_{12}\right)$	22.77%	18.27%	14.44%	17.95%
T300/LY556	$\left(\sigma_{11},\;\tau_{12}\right)$	22.81%	14.03%	22.02%	16.67%
E-glass/MY750	$\left(\sigma_{11}=\sigma_{33},\sigma_{22}\right)$	-	6.70%	14.92%	9.23%
IM7-8552	Off-axis compression	10.44%	9.73%	2.15%	8.77%
Glass/Epoxy	Off-axis compression	10.94%	10.80%	7.78%	10.10%

Note: For Figure 3a–c, the error calculation method is to draw a ray starting from the origin and connecting the experimental data points. The ratio of the distance between the experimental data and the envelope curve on the ray to the distance between the experimental data and the origin is the error of this point. For Figure 3d–f, the error calculation method is similar. Taking Figure 3d as an example, the ratio of the absolute value difference between the experimental data and the envelope curve at a certain value of $\sigma_{11}=\sigma_{33}$ to the absolute value of the experimental data at this point is the error of this point.

## Data Availability

The original contributions presented in this study are included in the article. Further inquiries can be directed to the corresponding author.
